# Using a large language model as a third reviewer to augment dual human full‐text screening in orthopaedic systematic reviews

**DOI:** 10.1002/ksa.70462

**Published:** 2026-05-20

**Authors:** Prushoth Vivekanantha, Helena Son, Luca Bernardini, Marc Daniel Bouchard, Olufemi Rolland Ayeni, Jeffrey Kay

**Affiliations:** ^1^ Division of Orthopedic Surgery, Department of Surgery McMaster University Hamilton Ontario Canada; ^2^ Michael de Groote School of Medicine McMaster University Hamilton Ontario Canada; ^3^ Princess Margaret Global Cancer Program Princess Margaret Cancer Centre Toronto Ontario Canada

**Keywords:** artificial intelligence, automation, full‐text, GPT‐5, large language model, systematic review

## Abstract

**Purpose:**

Large language models (LLMs) are a form of artificial intelligence (AI) that have emerged as potential tools to augment systematic review workflows. This study aimed to evaluate GPT‐5 as a third reviewer for full‐text screening across orthopaedic subspecialties.

**Methods:**

Three review topics were selected. Python scripts were developed to call on the GPT‐5 model via the OpenAI application programming interface (API) to perform full‐text screening using predefined inclusion and exclusion criteria. Two human reviewers simultaneously performed screening based on the same criteria. Performance metrics such as specificity, sensitivity, accuracy, positive predictive value (PPV), negative predictive values (NPV), and F1 scores for GPT‐5 were calculated based on a gold‐standard inclusion and exclusion list developed by a third human adjudicator. Efficiency metrics included total cost and completion time.

**Results:**

The number of full‐texts screened were 35, 70 and 146 amongst the three review topics. For topic one, sensitivity, specificity, PPV, NPV, accuracy and F1 scores were 100% each. For topic two, sensitivity, specificity, PPV, NPV, accuracy and F1 scores were 93.3%, 98.2%, 93.3%, 98.2%, 97.1% and 93.3% respectively. For topic three, sensitivity, specificity, PPV, NPV, accuracy and F1 scores were 93.3%, 100%, 100%, 99.2%, 99.3% and 96.7%, respectively. Time to completion ranged between 18.1 and 58 min. Cost ranged from $0.84 to $3.29 USD.

**Conclusion:**

GPT‐5 demonstrated high diagnostic accuracy as a third reviewer for full‐text screening across three different subspecialties, with high agreement with final consensus adjudication decisions. These findings suggest that modern LLMs can potentially augment dual‐review screening workflows by providing efficient decision‐support while preserving methodological rigour. However, the small number of included studies within each topic resulted in wide confidence intervals, and additional validation across larger datasets are necessary.

**Level of Evidence:**

Not applicable.

**Abbreviations**
AI: artificial intelligence
API: application programming interface
CI: confidence interval
FN: false negative
FP: false positive
GPT: Generative Pre‐trained Transformer
LLM: large language model
NLP: natural language processing
NPV: negative predictive value
PDF: portable document format
PPV: positive predictive value
RCT: randomised controlled trial
TAA: total ankle arthroplasty
TAR: total ankle replacement
TN: true negative
TP: true positive
USD: United States dollars

## INTRODUCTION

Systematic reviews are a core part of orthopaedic evidence as they are able to synthesise findings across a high volume of literature [5]. The use of orthopaedic reviews to inform practice, guidelines and decision‐making, stems from their unique ability to provide definitive answers to questions, and resolve discrepancies of conflicting results across studies [11]. A 2025 study identified that orthopaedic reviews have had an annual growth rate of 29.2% from 2001 to 2024, which demonstrates increasing demand for synthesised orthopaedic evidence [4]. However, as review volume increases, the reviewer burden and the risk of erroneous exclusion during study screening and adjudication becomes an important methodological concern.

Traditional models of machine learning have been explored to support systematic review screening throughout the 21st century, using text‐mining applications such as keywords, clustering and classifiers [13]. However, these methods have numerous limitations as they require significant author supervision, labelled training sets, limited applicability and risk of study generalisation [10]. Additionally, early machine learning models have typically been applied to title and abstract screening, with the aforementioned limitations posing a barrier to their application in full‐text screening [16]. However, with the emergence of large language models (LLMs), the avenue for having an AI‐based third reviewer may be more plausible.

In a previous study, a Python‐based natural language (NLP) script was developed to screen titles and abstracts using Generative Pre‐Trained Transformer‐5 (GPT‐5) via the OpenAI Application Programming Interface (API) [15]. This study demonstrated successful outputs with sensitivity ranging from 94.1% to 100.0%, specificity from 98.2% to 100.0%, positive predictive value from 92.3% to 100.0%, and negative predictive value from 99.8% to 100%, across all tasks [15]. This system demonstrated high performance in minimising FNs and was cost and time effective [15]. The robustness and operational efficiency of this model have served as strong supporting evidence to adjudicate its efficacy in full‐text screening, by screening entire texts, as opposed to just titles and abstracts. This can be particularly useful when inclusion or exclusion are not readily discernable from titles and abstracts alone, necessitating extensive full‐text adjudication and increasing the risk of erroneous exclusion by human reviewers.

This study aims to assess the efficacy and efficiency of GPT‐5 as a third reviewer for full‐text screening decisions across three review topics within three orthopaedic subspecialties, each with a distinct screening objective. It is hypothesised that compared to an adjudicated standard, GPT‐5 would have over 95% sensitivity and specificity.

## METHODS

### Study design and overview

Three distinct topics were chosen for the purpose of evaluating GPT‐5 as a third reviewer for full‐text screening in systematic reviews. The general workflow for methodological validation was adapted from a prior analysis assessing the utility of GPT‐5 for title and abstract screening [15]. To increase generalisability, each topic was within a distinct orthopaedic subspeciality (upper extremity, paediatrics and foot and ankle). No relevant reporting frameworks were followed.

### Data sources and full‐text sets

Initial comprehensive literature searches were performed on PubMed (Table 1). Alternative databases such as EMBASE or other databases were not used as the focus of this analysis was to evaluate the ability of LLMs as a third reviewer for adjudicating full‐text screening decisions, rather than to simulate the complete systematic review process. Title and abstract screening was then performed by GPT‐5 using a previously validated process [15]. GPT‐5 prompts for title and abstract screening are provided in Supporting Information: Appendix Table A. Prompts were designed a priori from the protocol eligibility criteria and applied in a zero‐shot manner without iterative refinement or modification during evaluation. The studies that met the inclusion criteria in the automated title and abstract screening were used for testing GPT‐5 in the full‐text stage.

**Table 1:** PubMed search strategies.

	Date searched	Search criteria
Topic 1: Upper extremity—Clinical outcomes after shoulder stabilisation for anterior shoulder instability or dislocations in skeletally immature patients	September 13, 2025	(pediatric OR skeletally immature OR skeletal immaturity OR adolescent) AND (bankart OR shoulder instability OR remplissage OR latarjet OR shoulder stabilisation)
Topic 2: Paediatric orthopaedics—studies reporting on non‐operative and operative management for paediatric medial epicondyle fractures of the humerus	October 13 2025	(medial epicondyle fracture OR medial epicondylar fracture OR medial humeral epicondyle OR medial epicondyle of humerus OR elbow medial epicondyle fracture OR (medial epicondyle AND fracture) OR (medial epicondylar AND fracture)) AND (pediatric OR pediatric OR children OR childhood OR adolescent OR teen OR teenage OR youth OR young))
Topic 3: Foot and ankle—Studies reporting on 5‐year post‐operative infection outcomes after total ankle arthroplasty	November 5 2025	(total ankle arthroplasty OR ankle arthroplasty OR ankle replacement OR total ankle replacement) AND (periprosthetic joint infection OR PJI OR wound infection OR infection OR deep infection OR wound complication OR superficial infection)

### Topic 1: Upper extremity

The first topic chosen was within the upper extremity subspecialty, namely, studies that reported on clinical outcomes after shoulder stabilisation surgery for anterior shoulder instability in skeletally immature patients. Initial search on PubMed was performed on 13 September 2025 and yielded 2747 eligible abstracts. Automated title and abstract screening resulted in 35 eligible full‐texts for review. Inclusion criteria for the full‐text stage included:

1. Human patients undergoing any form of shoulder stabilisation surgery for anterior shoulder instability.
2. Human patients who are described to be skeletally immature or to have open physes or open growth plates.
3. Studies reporting on clinical outcomes after surgical treatment for a skeletally immature group or subgroup.
4. Primary clinical research (randomised controlled trials, prospective cohorts, retrospective cohorts, case series).

Exclusion criteria were:

1. Studies with human patients that didn't have clinical outcomes reported for a skeletally immature group.
2. Patients without anterior shoulder instability and instead having either multi‐directional shoulder instability or posterior shoulder instability.
3. Systematic reviews, meta‐analyses, book chapters, editorials, commentaries, surgical technique papers without patient data and biomechanical, animal, or cadaveric studies.

### Topic 2: Paediatric orthopaedics

The second topic chosen was within the paediatric orthopaedic subspecialty and was on identifying studies reporting on outcomes after both non‐operative and operative management for medial epicondyle humerus fractures in patients under 18 years of age. Initial search on PubMed was performed on 13 October 2025, and yielded 471 eligible abstracts. Automated title and abstract screening resulted in 70 eligible full‐texts for review. Inclusion criteria for the full‐text stage included:

1. Patients under 18 years of age with medial epicondyle fracture of the humerus.
2. Studies that must report on clinical outcomes for both an operative and non‐operative group.

Exclusion criteria included:

1. Patients over the age of 18 with medial epicondyle fractures of the humerus.
2. Studies not reporting on clinical outcomes for both operative and non‐operative groups.
3. Systematic reviews, meta‐analyses, book chapters, editorials, commentaries, surgical technique papers without patient data and biomechanical, animal, or cadaveric studies.

### Topic 3: Foot and ankle

The third topic chosen was within the foot and ankle subspecialty, specifically, identifying studies reporting on infection outcomes after total ankle arthroplasty at the 5‐year follow‐up mark. Initial search on PubMed was performed on 5 November 2025 and yielded 1135 eligible abstracts. Automated title and abstract screening resulted in 146 eligible full‐texts for review. Inclusion criteria for the full‐text stage included:

1. Patients undergoing primary total ankle arthroplasty.
2. Infection outcomes reported at the 5 year follow‐up mark or if all patients had a minimum follow‐up of 5 years postoperatively.
3. Primary clinical research (randomised controlled trials, prospective cohorts, retrospective cohorts and case series).
4. Studies published in 2015 or later.

Exclusion criteria:

1. Patients undergoing procedures ankle arthrodesis without mention of a separate group undergoing total ankle arthroplasty.
2. Five‐year follow‐up infection outcomes not reported or studies with studies reporting on outcomes without specifying a minimum follow‐up of 5 years
3. Systematic reviews, meta‐analyses, book chapters, editorials, commentaries, surgical technique papers without patient data, and biomechanical, animal or cadaveric studies
4. Studies published prior to 2015 (2014 or before).

### Human review process

Two human reviewers independently screened each full‐text for eligibility based on the above‐mentioned inclusion and exclusion criteria on Covidence online software (Veritas Health Innovation, Melbourne, Australia). Discrepancies between the two human reviewers were flagged as conflicts. Pre‐conflict resolution inter‐rater agreement was calculated using Cohen's kappa with 95% confidence intervals (CIs).

### GPT‐5 screening

For each topic, portable document formats (pdfs) of eligible manuscripts from the full‐text stage were downloaded and organised into a folder. A python‐based natural language processing (NLP) script was developed by the primary author to screen full texts using GPT‐5 (OpenAI, San Francisco, California) via the OpenAI Application Programming Interface (API). The NLP script contains a 'prompt' that consists of instructions and inclusion and exclusion criteria for GPT‐5 to use to create decisions on each manuscript within the folder. GPT‐5 was also asked to provide a reason for exclusion for all studies that were excluded. Texts were extracted using the PyMuPDF library, a Python interface to the MuPDF document engine that retrieves text from digital pdfs. System prompts for full‐text screening can be found in Supporting Information: Appendix Table B. AI screening for the first, second, and third topics were performed on 23 September 2025, 3 November 2025, and 12 November 2025, respectively. All full texts per topic were performed in a single run. Full python scripts are provided as supplementary files for all three topics.

### Gold‐standard inclusion and exclusion decisions

Full‐texts included by both humans and GPT‐5 were assumed as correct, whether chosen to be included or excluded. Both did not undergo adjudication by the third senior human reviewer. All conflicts between human reviewers and all discrepancies between the LLM and humans were adjudicated by a more senior author. This was used to generate a list of gold‐standard inclusion and exclusion decisions to test GPT‐5 as the third reviewer.

### Model performance

Each inclusion and exclusion decision made by GPT‐5 was treated as a binary classification task: whether the decision was correctly or incorrectly included or excluded relative to the gold‐standard. True positives (TPs) were decisions where GPT‐5 correctly included the study, while decisions were reported as true negatives (TNs) if GPT‐5 correctly excluded the study. A false positive (FP) was considered when GPT‐5 incorrectly included a study, while a false negative (FN) was when GPT‐5 incorrectly excluded a study. These numbers were used to generate sensitivity, specificity, positive predictive value (PPV), NPV (negative predictive value), accuracy, and F1 score performance metrics. Performance metrics were reported as percentages and were calculated as follows for each review topic

PPV:TP/(TP+FP)×100NPV:TN/(TN+FN)×100Sensitivity:TP/(TP+FN)×100Specificity=TN/(TN+FP)×100Accuracy=(TP+TN)/(TP+FP+TN+FN)×100F1Score=2×(PPV×Recall)/(PPV+Recall).

CIs for proportions were calculated using Clopper–Pearson methods, which eliminates overshoot and zero width intervals, and yields conservative results [12]. CIs were not calculated for F1 scores as they are not binomial proportions.

### Efficiency outcomes

To assess efficiency and feasibility, runtime and API usage cost were tracked. The total time elapsed for parsing the pdfs of manuscripts and reporting on inclusion or exclusion decisions were recorded for each study. Similarly, API costs were recorded for each study. The sum of total cost and time for each study was then calculated to generate efficiency metrics for each review. API usage costs were calculated using OpenAI's available per‐token pricing for the GPT‐5 model. The equation was as follows: cost = (input tokens × 1.25/1,000,000) + (output tokens × 10/1,000,000). A token is the smallest unit of text that the model is able to process or generate.

## RESULTS

### Topic 1—Upper extremity

Of the 35 manuscripts screened, GPT‐5 classified nine as eligible for inclusion and excluded 26 studies. Humans included seven total studies before conflict resolution. There were five studies with conflicting decisions between the human reviewers, thus the pre‐resolution kappa was 0.646 (95%CI, 0.363–0.929).

Amongst the five human conflicts, three were correctly included by the AI model while two were correctly excluded. Therefore, amongst five studies with disagreement between the human reviewers, GPT‐5 matched the final adjudicated decision in all five cases (100%). Humans included one study that was excluded by the AI model. Upon adjudication, this study was identified to be incorrectly included by the humans. This study was Deitch et al. [3], which reported on patients with shoulder instability, however, outcomes for a distinct skeletally immature group undergoing surgery were not reported. There were no studies that GPT‐5 included that humans missed.

After final adjudication to create a gold‐standard inclusion and exclusion list, nine studies met the inclusion criteria and 26 were excluded. Based on this reference, GPT‐5 correctly classified all inclusions and exclusions, with nine TPs, 26 TNs, and zero FPs and FNs. The GPT‐5 performance metrics were 100% (95%CI 66.4%–100%), 100% (95%CI 86.8%–100%), 100% (95%CI 66.4%–100%), 100% (95%CI 86.8%–100%) and 100% (95%CI 90%–100%) for sensitivity, specificity, PPV, NPV and accuracy. The F1 score was 100%.The total cost to screen all 35 studies was $0.84 USD and the total time was 1,087.23 s (Table 2).

**Table 2:** GPT‐5 performance metrics.

	Total number of full‐texts	Sensitivity	Specificity	PPV	NPV	Accuracy	F1 score	Time to completion	Cost
Topic 1: Upper extremity—Clinical outcomes after shoulder stabilisation for anterior shoulder instability or dislocations in skeletally immature patients	35	100% (95%CI 66.4%–100%	100% (95%CI 86.8%–100%),	100% (95%CI 66.4%–100%)	100% (95%CI 86.8%–100%)	100% (95%CI 90%–100%)	100%	1,087.23 s (18.1 min)	$0.84USD
Topic 2: Paediatric orthopaedics —Studies reporting on non‐operative and operative management for paediatric medial epicondyle fractures of the humerus	70	93.3% (95%CI 68.1%–99.8%)	98.2% (95%CI 90.3%–100%)	93.3% (95%CI 68.1%–99.8%)	98.2% (95%CI 90.3%–100%)	97.1% (95%CI 90.1%–99.7%)	93.3%	1,987.55 s (33.1 min)	$1.58USD
Topic 3: Foot and ankle—Studies reporting on five‐year post‐operative infection outcomes after total ankle arthroplasty	146	93.3% (95%CI 68.1%–99.8%)	100% (95%CI 97.2%–100%)	100% (95%CI 76.8%–100%)	99.2% (95%CI 95.9%–100%)	99.3% (95%CI 96.2%–100%)	96.7%	3479.68 s (58 min)	$3.29USD

Abbreviations: CI, confidence interval; GPT‐5, Generative Pre‐Trained Transformer‐5; NPV, negative predictive value; PPV, positive predictive value; USD, United States Dollars.

### Topic 2—Paediatric orthopaedics

Of the 70 manuscripts screened, GPT‐5 classified 15 as eligible for inclusion and excluded 55 studies. Humans included 15 total studies before conflict resolution. There was only one study with conflicting decisions between the human reviewers, thus the pre‐resolution kappa was 0.961 (95%CI, 0.884–1.000).

The one human conflict was correctly included by the AI model (100%). Humans included two studies that were not included by the AI model. One was Axibal et al. [1], which was correctly excluded by the AI model, while the other was Kim et al. [8], which was incorrectly excluded. Axibal et al. [1] while correctly comparing outcomes for non‐operative and operative treatment of medial epicondyle fractures, this study also included patients who were 19 years of age. The decision reason for excluding Kim et al. [8] was 'No information in abstract, methods, results or tables to confirm paediatric population, comparative treatments, outcomes and primary study design'. GPT‐5 also incorrectly included one study that was not included by humans. This study was McGraw‐Heinrich et al. [9], which reported on patients with concomitant supracondylar humerus fractures without mention of a non‐operative management group.

After final adjudication to create a gold‐standard inclusion and exclusion list, 15 studies met the inclusion criteria and 55 were excluded. Based on this reference, GPT‐5 had 14 TPs, 54 TNs, one FP and one FN. Thus, performance metrics for sensitivity, specificity, PPV, NPV, accuracy were 93.3% (95%CI 68.1%–99.8%), 98.2% (95%CI 90.3%–100%), 93.3% (95%CI 68.1%–99.8%), 98.2% (95%CI 90.3%–100%), 97.1% (95%CI 90.1%–99.7%), respectively. The F1 score was 93.3%. The total cost to screen all 70 studies was $1.58 USD and the total time was 1987.55 s (Table 2).

### Topic 3—Foot and Ankle

Of the 146 manuscripts screened, GPT‐5 classified 14 as eligible for inclusion and excluded 132 studies. Humans included 14 total studies before conflict resolution. There was only one study with conflicting decisions between the human reviewers, thus the pre‐resolution kappa was 0.962 (95%CI, 0.887–1.000).

The one human conflict was correctly included by the AI model (100%). Humans included two studies that were not included by the AI model. One was Kim et al. [7], which was incorrectly excluded by the AI model, while the other was Dagneaux et al. [2], which was correctly excluded. The reason provided by GPT‐5 for excluding Kim et al. [7] was ‘Infection outcomes at or beyond five years for the primary TAA cohort are not reported in the abstract, methods, results, or tables.’ Dagneaux et al. [2] did not report on infection outcomes specifically at the 5‐year mark or beyond and also had patients with latest follow‐up times of less than 5 years post‐operative. There was one study that was correctly identified by the AI that was missed by the humans.

After final adjudication to create a gold‐standard list inclusion and exclusion list, there were 15 correct includes and 131 correct excludes. Based on this reference, GPT‐5 had 14 TPs, 131 TNs, zero FPs and one FN. Thus, the sensitivity, specificity, PPV, NPV and accuracy values were 93.3% (95%CI 68.1%–99.8%), 100% (95%CI 97.2%–100%), 100% (95%CI 76.8%–100%), 99.2% (95%CI 95.9%–100%), 99.3% (95%CI 96.2%–100%), respectively. The F1 score was 96.7%. The total cost to screen all 146 studies was $3.29 USD and the total time was 3479.68 s (Table 2).

## DISCUSSION

GPT‐5 demonstrated high diagnostic accuracy performance for automated full‐text screening across three orthopaedic subspecialties, with consistently high accuracy, sensitivity and specificity. Performance was high for the upper extremity review, and remained high for both the paediatric and foot‐and‐ankle topics. These findings indicate that GPT‐5 may be able to generalise full‐text screening accuracy across distinct orthopaedic clinical domains, including shoulder instability, paediatric elbow fractures, and total ankle arthroplasty infection outcomes. Importantly, this level of performance was achieved with minimal computational burden: all three reviews were completed in 18–58 min at a cost of only $0.84–$3.29 USD. Together, these results demonstrate that modern LLMs can serve as a third reviewer and decision support tool for authors to minimise erroneous exclusions.

Prior studies evaluating LLM‐based full‐text screening in medicine have generally reported near‐human performance but often with modest drops in sensitivity or specificity when applied to complex clinical manuscripts [14]. Existing evaluations in general medicine, oncology and rehabilitation have shown that LLMs can approximate human‐level inclusion decisions but frequently require extensive prompt tuning and still produce non‐negligible amounts of FNs [6]. Within that context, the consistently high performance of GPT‐5 across three orthopaedic subspecialties exceeds what has been reported in earlier‐generation models. Moreover, these findings extend prior work validating GPT‐5 for title‐and‐abstract screening, which also demonstrated good performance (e.g., sensitivity 94.1%–100.0%, specificity 98.2%–100.0%, PPV 92.3%–100.0%; NPV 99.8%–100.0%; F1 scores 95.9%–97.0% across all tasks) [15]. As full‐text screening requires interpreting dense methodological details, population definitions, and nuanced outcome reporting, it represents a substantially more difficult task than abstract screening alone. These results demonstrate that LLMs can support authors with abstract and full‐text screening to enhance their systematic reviews and improve methodological rigour.

The small number of errors observed across topics highlights the central role of prompt engineering in LLM‐based screening. All misclassifications and performance errors were attributable to predictable interactions between prompt structure and topic‐specific edge cases. In the paediatric review, the FP included concomitant supracondylar fractures, which fit the surface pattern of 'operative + non‐operative paediatric elbow fracture study', but did not meet the protocol's requirement for isolated medial epicondyle fractures [9]. As the prompt did not explicitly instruct the model to exclude studies with concomitant injuries, GPT‐5 interpreted the manuscript as eligible. Conversely, the FN arose from missing or insufficiently explicit statements in the allowed sections regarding paediatric age or stratified outcomes, leading GPT‐5 to treat required information as ‘not reported’ per the protocol [8]. This suggests that eligibility‐relevant information was not detectable within the extracted text available to the model, highlighting a limitation of automated full‐text processing in which formatting‐dependent content may not be reliably captured during pdf parsing. A similar mechanism explains the single FN in the ankle arthroplasty review, where follow‐up duration and infection‐specific timepoints were ambiguously linked in the text [7]. These cases underscore that while LLMs can execute protocol‐faithful reasoning with remarkable precision, optimal prompt construction, including explicit handling of edge cases, concomitant injuries, mixed cohorts and follow‐up logic, remains an open area for systematic investigation.

These findings support the role of LLMs as a screening adjunct or third reviewer on a prefiltered set under a specific pipeline. Rather than functioning as a replacement for human judgement, the model demonstrated concordance with adjudicated decisions and can aid in cases of human disagreement, as noted by the conflicts that arose between human reviewers. LLMs can help identify studies that humans missed and vice versa, as highlighted by the foot and ankle review simulation. The LLM missed one study that the humans correctly included but it also included one study that the humans incorrectly excluded. With 15 studies eligible as per the inclusion and exclusion criteria for that review, even missing one study can result in clinically significant and misleading meta‐analytic conclusions. Thus, the use of AI offers an appealing solution to maximise catchment for full‐text screening. However, it is important that this technology should be validated under a fully independent reference standard before being positioned as a safety‐critical reviewer.

Several limitations warrant consideration. First, the study evaluated GPT‐5 using PDFs from a single database and within predefined subspecialties; performance may differ for more heterogeneous or lower‐quality manuscripts, non‐English articles, or topics with more ambiguous inclusion criteria. Second, success of the AI was likely not only due to LLM reasoning, but also likely due to variability in manuscript organisation. Additionally, the prompt structures used here, though effective, represent only one of many possible strategies, and different prompt formulations may yield different sensitivity‐specificity tradeoffs. Another limitation is that the study suffers from partial verification bias given that agreements between humans and the AI were not adjudicated. The difference between the reference standard and the true list for inclusions and exclusions may be present, potentially inflating performance estimates. The gold‐standard was also not an objective external standard but instead via adjudication by a senior human reviewer which embeds human sensitivity. This study also was performed exclusively on PubMed‐derived records, which deviates from standard systematic review methodology which includes multiple databases, thus performance estimates may be inflated. However, this study was meant to be a proof‐of‐concept rather than a full simulation of real‐world workflows. Fourth, the full‐text set was pre‐selected by GPT‐5 which performed a title and abstract screening, which risks selection bias and may inflate or deflate downstream full‐text performance. Finally, as with all LLM applications, unpredictable errors may occur, hence why these tools should be used as decision‐support agents rather than replacements.

## CONCLUSION

GPT‐5 demonstrated high diagnostic accuracy as a third reviewer for full‐text screening across three different subspecialties, with high agreement with final consensus adjudication decisions. These findings suggest that modern LLMs can potentially augment dual‐review screening workflows by providing efficient decision‐support while preserving methodological rigour. However, the small number of included studies within each topic resulted in wide confidence intervals, and additional validation across larger datasets are necessary.

## AUTHOR CONTRIBUTIONS

**Prushoth Vivekanantha**: Idea conception; code generation; data analysis; writing; editing. **Helena Son**: Human reviewer; writing; editing. **Luca Bernardini**: Human reviewer; writing; editing. **Marc Daniel Bouchard**: Writing; editing; reviewing. **Olufemi Rolland Ayeni**: Writing; editing; reviewing; supervision. **Jeffrey Kay**: Writing; editing; reviewing; supervision.

## CONFLICT OF INTEREST STATEMENT

Olufemi Rolland Ayeni reports that they are part of the Speakers Bureaum for Stryker Canada and that they are the owner of Notch Academy. The remaining authors declare no conflicts of interest.

## ETHICS STATEMENT

The authors have nothing to report.

## Supporting information


Supporting File

## Data Availability

Data can be made available upon reasonable request at prushoth.vivekanantha@medportal.ca.
