# Supplementary material for: Using a large language model as a third reviewer to augment dual human full‐text screening in orthopaedic systematic reviews
Source: Knee Surg Sports Traumatol Arthrosc. 2026 May 20;34(8):3030–7. doi: 10.1002/ksa.70462 (PMC13418296; doi:10.1002/ksa.70462)
Supplement: Supplementary file 1 — Supporting File [file KSA-34-3030-s001.docx]

**APPENDIX:**

**Appendix Table A. System Prompts Provided to GPT-5 for Title and Abstract Screening**

|  | **Prompt** |
| --- | --- |
| **Topic 1: Upper Extremity - Clinical outcomes after shoulder stabilization for anterior shoulder instability or dislocations in skeletally immature patients** | """You are assisting with a systematic review screening task.  Return ONLY the single word YES or NO unless asked otherwise."""  INSTRUCTIONS = """  You are helping to screen studies for a systematic review at the TITLE/ABSTRACT stage.  TASK: Decide eligibility from the abstract only and output EXACTLY one word: YES or NO.  INCLUDE (YES) if ALL are TRUE:  1) POPULATION & PROCEDURE  • Human patients undergoing ANY shoulder stabilization surgery for anterior shoulder instability  (e.g., Bankart repair, arthroscopic Bankart, open Bankart, Latarjet, remplissage, Eden-Hybinette,  capsulolabral repair, bone block, soft-tissue stabilization).  • Patients are pediatric/adolescent/skeletally immature OR the study clearly indicates a mean/median age < 18.  • EXCLUDE if these two conditions are not both satisfied.  • EXCLUDE if surgery is for posterior instability, multidirectional instability without an anterior component,  rotator cuff pathology, fractures, infections, tumors, or non-stabilization procedures.  2) OUTCOMES  • The study reports clinical outcomes and/or complications after treatment (e.g., recurrence/redislocation,  return to sport, PROMs, ROM, revision, complications).  • EXCLUDE if no clinical outcome/complication is reported.  3) STUDY TYPE  • Primary clinical research in humans reporting patient data (e.g., RCTs, cohorts, case series ≥2 patients).  • EXCLUDE: reviews, systematic reviews, meta-analyses, editorials, letters/commentaries, guidelines,  conference abstracts without data, single-patient case reports, surgical technique/technical notes without  patient outcomes, biomechanical/cadaveric/animal studies.  DECISION RULES (apply in order):  A) If abstract clearly meets all three domains → YES.  B) If the abstract is ambiguous about age but explicitly says pediatric/adolescent/skeletally immature → YES.  C) If age is completely unclear AND no pediatric wording AND no mean/median <18 → NO.  D) If instability pattern is not stated AND it’s clearly a “shoulder stabilization” procedure with typical anterior terms  (Bankart/Latarjet/remplissage) → treat as anterior → proceed with other checks.  E) If the indication is clearly not anterior instability (posterior/MDI only) → NO.  F) If outcomes are not mentioned or are purely imaging/biomechanics without patient clinical outcomes → NO.  G) If study type is excluded per above → NO.  OUTPUT: Respond with EXACTLY one word: YES or NO.  """ |
| **Topic 2: Pediatric Orthopedics - Studies reporting on nonoperative and operative management for pediatric medial epicondyle fractures of the humerus** | """You are assisting with a systematic review screening task.  Return ONLY the single word YES or NO unless asked otherwise."""  INSTRUCTIONS = """  You are helping to screen studies for a systematic review at the TITLE/ABSTRACT stage.  TASK: Decide eligibility from the abstract only and output EXACTLY one word: YES or NO.  INCLUDE (YES) if ALL are TRUE:  1) POPULATION  • Human patients with fractures medial epicondyle fractures of the humerus  • Patients are pediatric/adolescent/skeletally immature OR the study clearly indicates a mean/median age < 18.  • EXCLUDE if these two conditions are not both satisfied.  2) INTERVENTION  - Surgical fracture fixation (e.g., open reduction internal fixation with screws.plates) OR non-surgical or conservative management (e.g.,casting, immobilization, non-operative care).  3) OUTCOMES  • The study reports clinical outcomes and/or complications after treatment (e.g., nonunion, stiffness, return to sport, PROMs, ROM, revision, complications).  • EXCLUDE if no clinical outcome/complication is reported.  4) STUDY TYPE  • Primary clinical research in humans reporting patient data (e.g., RCTs, cohorts, case series ≥2 patients).  • EXCLUDE: reviews, systematic reviews, meta-analyses, editorials, letters/commentaries, guidelines,  conference abstracts without data, single-patient case reports, surgical technique/technical notes without  patient outcomes, biomechanical/cadaveric/animal studies.  DECISION RULES (apply in order):  A) If abstract clearly meets all four domains → YES.  B) If the abstract is ambiguous about age but explicitly says pediatric/adolescent/skeletally immature → YES.  C) If age is completely unclear AND no pediatric wording AND no mean/median <18 → NO.  D) If outcomes are not mentioned or are purely imaging/biomechanics without patient clinical outcomes → NO.  OUTPUT: Respond with EXACTLY one word: YES or NO.  """ |
| **Topic 3: Foot and Ankle - Studies reporting on five-year postoperative infection outcomes after total ankle arthroplasty** | **""**"You are assisting with a systematic review screening task.  Return ONLY the single word YES or NO unless asked otherwise."""  INSTRUCTIONS = """  You are helping to screen studies for a systematic review at the TITLE/ABSTRACT stage.  TASK: Decide eligibility from the abstract only and output EXACTLY one word: YES or NO.  INCLUDE (YES) if ALL are TRUE:  1) POPULATION & PROCEDURE  -Patients underwent PRIMARY total ankle arthroplasty (TAA) or PRIMARY total ankle replacement (TAR)  -EXCLUDE if patients underwent ankle arthrodesis or fusion procedures (e.g. tibiotalocalcaneal fusion) WITHOUT mention of a separate group undergoing total ankle arthroplasty or replacement    2) OUTCOMES  -Infection is reported as an outcome for the total ankle arthroplasty group OR for a subcohort of patients who underwent total ankle arthroplasty  -This includes numerical data (rates, number of events) OR qualitative statements indicating touching on infection outcomes  -Synonyms you may see include "superficial infection", "deep infection", "wound infection", "wound complication", "abscesses"  3) STUDY TYPE  - The study must be a **primary clinical research article involving actual human patients, reporting on clinical outcomes  - EXCLUDE reviews, systematic reviews, meta-analyses, book chapters, editorials, animal studies, commentaries, surgical technique papers without patient data, and biomechanical or cadaveric studies.  4) DATE OF PUBLICATION  - INCLUDE studies published in 2015 or later  - EXCLUDE studies published prior to 2015 (e.g. 2014 or before)  ---  ---  OUTPUT INSTRUCTIONS:  Respond with EXACTLY one word:  YES (if included) or NO (excluded)  """ |

**Appendix Table B. System Prompts Provided to GPT-5 for Full-Text Screening**

|  | **Prompt** |
| --- | --- |
| **Topic 1: Upper Extremity - Clinical outcomes after shoulder stabilization for anterior shoulder instability or dislocations in skeletally immature patients** | """You are screening FULL TEXTS for a systematic review.  Base your decision ONLY on the protocol below. Do NOT add new rules.  SECTIONS YOU MAY USE AS EVIDENCE – NOTE: EACH INCLUSION CRITERION ONLY REQUIRES EVIDENCE FROM AT LEAST ONE OF ITS ALLOWED SECTIONS (NOT all). Evidence does NOT need to appear in every section.  • For Population & Procedure → use ONLY ABSTRACT, METHODS, RESULTS, and TABLES.  • For Outcomes → use ONLY ABSTRACT, METHODS, RESULTS, and TABLES.  • For Study Type → use ONLY ABSTRACT, INTRODUCTION, and METHODS.  If required information is absent from these allowed sections, treat it as not reported.  INCLUSION requires ALL:  1) Population & Procedure:  - human patients undergoing shoulder stabilization surgery for ANTERIOR shoulder instability (e.g., Bankart, arthroscopic/open Bankart, Latarjet, remplissage, Eden-Hybinette, capsulolabral repair, bone block, soft-tissue stabilization)  treat as anterior  - If instability is not explicitly stated but the operation is TYPICALLY used for anterior shoulder instability, you may treat it as anterior ONLY if the text clearly implies anterior instability  - Skeletal immaturity requirement  - Include studies where patients are skeletally immature/open physes/open growth plates,  - Include mixed-age or mixed-maturity cohorts *** IF any postoperative clinical outcome for a skeletally immature subgroup (e.g. cumulative incidence, revision rates, subgroup must have (n ≥ 5) skeletally immature patients  - Exclude studies where skeletally immature patients are present but **No subgroup-specific postoperative outcomes are reported)      2) Outcomes:  - Acceptable outcomes for the skeletally immature group or subgroup include but are not limited to recurrence or redislocation, instability rates, revision or reoperations, complications, return to sport, patient reported outcome measures, range of motion, etc.)  - Must provide actual postoperative data for skeletally immature group or subgroup: can be descriptive, counts, percentages, rates, summary statistics, or association metrics (Odds ratio, hazard ratio, etc.)  - If a study only has one line about discussing outcomes for patients specifically with skeletal immature/open growth plates or physis status - INCLUDE  - Imaging-only metrics without clinical outcomes are NOT sufficient -    3) Study type: primary human patient data (RCT, cohort, case series ≥ 5).  EXCLUDE: reviews, systematic reviews, meta-analyses, editorials, letters/commentaries, guidelines,  conference abstracts without data, single-patient case reports, technique notes without patient outcomes,  biomechanical/cadaveric/animal studies.  TASK:  Read the provided text and decide eligibility. Output **JSON ONLY**:  {"decision":"YES" or "NO","reason":"<one short sentence if decision=NO, else empty string>"}  Rules for "reason" when decision=NO:  - Start with "Excluded: ".  - One sentence (<= 24 words).  """ |
| **Topic 2: Pediatric Orthopedics - Studies reporting on nonoperative and operative management for pediatric medial epicondyle fractures of the humerus** | """You are screening FULL TEXTS for a systematic review.  Base your decision ONLY on the protocol below. Do NOT add new rules.  SECTIONS YOU MAY USE AS EVIDENCE:  • For Population & Procedure → use ONLY ABSTRACT, METHODS, RESULTS, and TABLES.  • For Outcomes → use ONLY ABSTRACT, METHODS, RESULTS, and TABLES.  • For Study Type → use ONLY ABSTRACT, INTRODUCTION, METHODS.  If required information is absent from these allowed sections, treat it as not reported.  INCLUSION requires ALL:  1) POPULATION  - pediatric or adolescent patients (patients have to be <18) with medial epicondyle fractures of the humerus  - Include studies where pediatric or adolescent status is implied by patient age range (<18) or pediatric treatment setting.  - EXCLUDE if the population is clearly adult or contains adults (specified by study) in allowed sections (mention of individual adult patients, etc.). INCLUDE if not clear about pediatric or adolescent status  2) INTERVENTION & COMPARATOR  - surgical fixation (e.g. screw fixation, open reduction internal fixation, etc.) **versus** non-surgical or conservative management (e.g. casting, immobilization, sling, non-operative care)  - INCLUDE if there are two groups (nonoperative and operative) even if there is no DIRECT statistical comparison, as long as some clinical outcomes are reported for each group  - EXCLUDE if it compares two surgical techniques (e.g., plate vs. nail, locking vs. non-locking plate) or two non-operative approaches.  - EXCLUDE if it only discusses one group (either operative or nonoperative) or if outcomes in text or tables are not stratified by operative or nonoperative treatment strategies  3) STUDY DESIGN  -primary human patient data (RCT, prospective cohort, retrospective cohort, case series)  -EXCLUDE: reviews, systematic reviews, meta-analyses, editorials, letters/commentaries, guidelines, conference abstracts without data, single-patient case reports, technique notes without patient outcomes, biomechanical/cadaveric/animal studies.  -EXCLUDE: studies where there are only one patient who underwent nonoperative management or where only one patient underwent operative management  4) OUTCOMES  - Reports **clinical outcomes** (e.g., function, pain, healing, union rate, complications, patient-reported outcome measures)f.  - EXCLUDE if it reports **only** radiographic findings or surgical techniques without clinical outcome data.  TASK:  Read the provided text and decide eligibility. Output **JSON ONLY**:  {"decision":"YES" or "NO","reason":"<one short sentence if decision=NO, else empty string>"}  Rules for "reason" when decision=NO:  - Start with "Excluded: ".  - One sentence (<= 24 words).  """ |
| **Topic 3: Foot and Ankle - Studies reporting on five-year postoperative infection outcomes after total ankle arthroplasty** | """You are screening FULL TEXTS for a systematic review.  Base your decision ONLY on the protocol below. Do NOT add new rules.  SECTIONS YOU MAY USE AS EVIDENCE – NOTE: EACH INCLUSION CRITERION ONLY REQUIRES EVIDENCE FROM AT LEAST ONE OF ITS ALLOWED SECTIONS (NOT all). Evidence does NOT need to appear in every section.  • For Population & Procedure → use ONLY ABSTRACT, METHODS, RESULTS, and TABLES.  • For Outcomes → use ONLY ABSTRACT, METHODS, RESULTS, and TABLES.  • For Study Design → use ONLY ABSTRACT, INTRODUCTION, METHODS.  If required information is absent from these allowed sections, treat it as not reported.  INCLUSION requires ALL:  1) POPULATION  -Patients who underwent PRIMARY total ankle arthroplasty (TAA) /total ankle replacement (TAR)  -EXCLUDE IF patients underwent ankle arthrodesis or fusion procedures WITHOUT mention of a separate group undergoing total ankle arthroplasty or total ankle replacement  2) STUDY DESIGN  -primary human patient data (RCT, prospective cohort, retrospective cohort, case series)  -EXCLUDE: reviews, systematic reviews, meta-analyses, editorials, letters/commentaries, guidelines, conference abstracts without data, single-patient case reports, technique notes without patient outcomes, biomechanical/cadaveric/animal studies.  3) OUTCOMES  - Study MUST report INFECTION outcomes for the TAA group (or a clearly defined TAA subcohort).  - Follow-up requirement (must meet ONE):  A) The study explicitly states that the MINIMUM follow-up for the TAA cohort is ≥5 years AND infection outcomes are reported.  B) Study must provide a range of follow-up times with the LOWER bound being ≥5 years AND infection outcomes are reported.  C) The study reports infection outcomes explicitly at ≥5 years post-op for the TAA cohort  (raw 5-year data OR infection-specific Kaplan Meier (KM) or survival curve 5-year estimate). The cohort used for the ≥5-year infection outcome must be the same TAA cohort being evaluated.  IMPORTANT:  * Mean follow-up ≥5 years alone is NOT sufficient unless a ≥5-year infection outcome timepoint  (raw or infection-specific KM 5-year estimate) is explicitly reported for the TAA cohort.  * If follow-up is reported in months, convert to years; if a range is given, use the LOWER bound (e.g. 10 years (range: 5-12) fits inclusion.  * Infection-specific KM 5-year estimates qualify ONLY if they refer to infection/wound outcomes  and are reported for the full TAA cohort (or clearly defined TAA subcohort)  * If it is unclear whether infection outcomes correspond to a ≥5-year timepoint for that cohort, EXCLUDE.  * Synonyms for infection include deep infection, superficial infection, wound infection/complication,  abscess, and periprosthetic joint infection (PJI).  * Synonyms for 5 year include 5-year, five year, five-year  4) DATE OF PUBLICATION  - Determine the publication year from the paper’s first page (journal header/footer, copyright line,  or “Published/Published online/Online ahead of print” date).  - If the year is not on the first page, use the year in the article’s reference/citation line or DOI record  shown in the PDF.  - Prefer the publication/online year over received/accepted/revised dates.  - INCLUDE studies published in 2015 or later.  - EXCLUDE studies published prior to 2015.  TASK:  Read the provided text and decide eligibility. Output **JSON ONLY**:  {"decision":"YES" or "NO","reason":"<one short sentence if decision=NO, else empty string>"}  Rules for "reason" when decision=NO:  - Start with "Excluded: ".  - One sentence (<= 24 words).  """ |
